# How can healthcare professionals provide guidance and support to parents of adolescents? Results from a primary care-based study

**DOI:** 10.1186/s12913-021-06200-1

**Published:** 2021-03-20

**Authors:** Lindsey D. Jones, Randall W. Grout, Amy L. Gilbert, Tracey A. Wilkinson, Tamila Garbuz, Stephen M. Downs, Matthew C. Aalsma

**Affiliations:** 1 Department of Psychology, Indiana University-Purdue University Indianapolis, 402. N Blackford Street, LD 120B, Indianapolis, IN 46202 USA; 2 Department of Pediatrics, Indiana University School of Medicine, 410 W. 10th Street, Suite 2000, Indianapolis, IN 46202 USA; 3 Department of Environmental Health and Safety, Indiana University-Purdue University Indianapolis, 980 Indiana Ave Room 4425, Indianapolis, IN 46202 USA; 4 Center for Biomedical Informatics, Wake Forest University School of Medicine, 486 N. Patterson Ave, Winston-Salem, NC 27101 USA; 5 Department of Pediatrics, Indiana University School of Medicine, 410 W. 10th Street, Suite 2025, Indianapolis, IN 46202 USA

**Keywords:** Parents, Adolescents, Primary care, Parenting support

## Abstract

**Background:**

This study explored the rewards and difficulties of raising an adolescent and investigated parents’ level of interest in receiving guidance from healthcare providers on parenting and adolescent health topics. Additionally, this study investigated whether parents were interested in parenting programs in primary care and explored methods in which parents want to receive guidance.

**Methods:**

Parents of adolescents (ages 12–18) who attended an outpatient pediatric clinic with their adolescent were contacted by telephone and completed a short telephone survey. Parents were asked open-ended questions regarding the rewards and difficulties of parenting and rated how important it was to receive guidance from a healthcare provider on certain parenting and health topics. Additionally, parents reported their level of interest in a parenting program in primary care and rated how they would like to receive guidance.

**Results:**

Our final sample included 104 parents, 87% of whom were interested in a parenting program within primary care. A variety of parenting rewards and difficulties were associated with raising an adolescent. From the list of parenting topics, communication was rated very important to receive guidance on (65%), followed by conflict management (50%). Of health topics, parents were primarily interested in receiving guidance on sex (77%), mental health (75%), and alcohol and drugs (74%). Parents in the study wanted to receive guidance from a pediatrician or through written literature.

**Conclusions:**

The current study finds that parents identify several rewarding and difficult aspects associated with raising an adolescent and are open to receiving guidance on a range of parenting topics in a variety of formats through primary care settings. Incorporating such education into healthcare visits could improve parents’ knowledge. Healthcare providers are encouraged to consider how best to provide parenting support during this important developmental time period.

**Supplementary Information:**

The online version contains supplementary material available at 10.1186/s12913-021-06200-1.

## Background

Adolescence is a period when teens increasingly separate from their parents and begin to establish autonomy [1], and thus, parents find the transition from childhood to adolescence as a difficult time to parent [2]. Parents report a decrease in parenting satisfaction during adolescent development, [3] which is due to families experiencing increased parent-adolescent conflict, stress, and emotional distance [4]. Because parenting skills change as youth develop from childhood to adolescence, parents can develop low parental self-esteem and self-doubt [3] - a sentiment which can lead to adverse parenting styles and place adolescents at an increased risk for unhealthy development [5]. Knowing that parents play a pivotal role in the maturation of their adolescents’ health, approaches for providing support to parents is critical. One possible approach to providing parental support is through healthcare provider (HCP) anticipatory guidance (brief, office-based feedback to parents from HCPs) and provision of parenting programs (structured curriculum provided to parents either in primary care or community settings with the use of technology or in-person consultations) [6, 7].

Strong partnerships between HCPs and parents can positively impact adolescent health outcomes [8, 9]. The American Academy of Pediatrics (AAP) recognizes this by recommending parents and adolescents each meet privately with HCPs [10]. Separation during healthcare appointments allots time for parents to receive anticipatory guidance from HCPs, while also granting opportunities to discuss individual needs (e.g., advice on adolescent behaviors, mood changes, and puberty). Evidence finds that adolescents want to receive health information from HCPs and parents [11]; therefore, anticipatory guidance not only improves parent knowledge and self-efficacy, but also provides comfort to adolescents who may feel more inclined to speak privately with their parent about their health concerns. Moreover, the implementation of evidence-based parenting programs in primary care could provide additional resources to support, educate, and enhance parenting skills [12]. Although healthcare systems have yet to incorporate universal parenting programs into practice, adoption of such programs could provide opportunities for parents seeking information beyond brief consultations with HCPs providing anticipatory guidance [13]. Considering parents already report viewing HCPs as reliable resources for preventative adolescent *health topics* [14–16]; it is important to understand whether parents are interested in seeking support from HCPs on *parenting topics*. Specifically, research has yet to explore parents’ perspectives of receiving support and guidance from HCP’s on practices that influence adolescent risky behavior like discipline, parental monitoring, and conflict management [17].

The majority of parenting programs are found in non-medical settings, [18–20] and strive to modify parenting practices through the use of behavioral and social learning theories [21]. In general, parenting programs effectively change behaviors [19, 21] and are successful in improving family conflicts, parenting practices, and adolescent behavioral and mental health [22, 23]. Despite evidence that parenting programs improve adolescent health, [20] increase positive parenting practices, [22, 23] and reduce economic costs, [24, 25] support programs for parents of adolescents have yet to be integrated into universal practice in healthcare settings. Instead, the majority of parenting programs in healthcare settings are centered on serving parents of young children (e.g., early family educators, birthing classes, assistance programs for childhood behavioral problems), not adolescents [18, 26–28]. Given that parents use support programs in non-medical settings, and parents already find HCPs a trusting resource for information, it is important to understand whether parents are interested in receiving *parenting* support and guidance in a healthcare setting.

The present study’s purpose was to: (1) explore the rewards and difficulties associated with parenting an adolescent (2) identify what *parenting topics* would be of most interest to receive guidance on from HCPs and (3) determine if a primary care-based parenting program would be of interest to parents. The secondary aims were to (1) determine the most appropriate method of delivering anticipatory guidance to parents and (2) extend previous literature by re-examining health topics parents find important to discuss with HCPs.

## Methods

### Recruitment and procedures

This project was secondary to a larger study examining the utilization of a clinical decision support system (CDSS) - called the Child Health Improvement through Computer Automation System (CHICA) - to evaluate the system’s impact on identifying adolescent physical and mental health problems and improving primary care management of adolescent health. CHICA is a CDSS that integrates electronic medical record data (e.g., diagnoses, health risks, demographics), pre-visit screening data (e.g., scores on depression, anxiety, or substance use measures), and responses from previous healthcare visits to generate appropriate follow-up recommendations, tools, and prompts for HCPs prior to meeting with a patient [29]. The present study used information gathered from the CHICA system to generate a list of parents and adolescents (ages 12 to 18) who recently presented to their pediatric primary care clinic and who completed an electronic-based screening tool prior to the outpatient visit.

Data were pulled from five participating primary care clinics; all clinics were from the same hospital system and predominately served patients from socioeconomically disadvantaged backgrounds. Using extracted data from the CHICA system, lists of eligible participants were generated daily based on families who attended a healthcare appointment the day prior. Once the list of eligible participants was generated, the Indiana University Pediatric Research Network (PResNet) team pre-populated adolescent and parent contact information into a secure electronic research database (REDCap). Research assistants (LJ, TG, EC) were given 48 h – 2 days after the pediatric primary care visit - to contact parents by telephone and invite them to participate in a short eligibility screen and survey. Eligible parents had to have (1) accompanied their adolescent to the pediatric primary care visit, (2) were the caregiver or legal guardian of the adolescent who attended the healthcare appointment, and (3) completed - or whose adolescent completed – at least 10 questions on the CHICA pre-screener prior to meeting with the pediatric HCP. Upon completing the eligibility screen, parents were invited to participate in a telephone survey about their experiences with raising an adolescent and their thoughts on receiving anticipatory guidance and parenting support in primary care. Recruitment occurred between June and August of 2017 and concluded once a predetermined sample size (based on the results of a similar study [16]), was achieved. Verbal consent was collected from all study participants prior to beginning the survey. After completing the survey, parents received a $25 gift certificate to compensate them for their time. This study was classified and operated under exempt approval by the Indiana University Human Subjects Office.

### Demographics

Adolescent demographic information (i.e., age, race, ethnicity, sex) was collected by drawing data from the CHICA system and integrating it into REDCap. Parent demographic information was collected over the phone by a research assistant, prior to starting the telephone survey.

#### Measures

Survey data were obtained using a series of items. All survey items were administered over the telephone by research staff (LJ, TG, EC) who were fluent in English and/or Spanish. The survey took no more than 30 min to complete. Survey items are described below based on topic.

### Rewards and difficulties of parenting an adolescent

Parents were asked two open-ended questions about their experiences raising an adolescent: (1) what are the rewards of parenting and (2) what are the difficulties of parenting. Responses were recorded verbatim by research staff.

### Parenting program

Parents were asked whether they would participate in a parenting support program if it were offered to them by their primary care clinic or HCP. Reponses were coded simply as: (1) yes or (2) no.

### Parenting topics

A 4-item measure was developed by research staff based on literature discussing typical challenges faced by parents during adolescent development [30]. Parents of adolescents were asked to rate which of the following parenting topics would be important to receive anticipatory guidance on from a HCP: (1) conflict management, (2) discipline, (3) parental monitoring, and (4) communication. Level of importance for each chosen topic was measured using a 3-point rating scale with options of: *(1) very important, (2) somewhat important* and *(3) not at all important.*

### Health topics

A 12-item adapted measure was used to assess which adolescent health topics parents perceived as important to discuss with a HCP [16, 31]. Parents could choose multiple answers from the following topics: (1) romantic partners, (2) friendships, (3) internet use, (4) use of mobile technology (e.g., cell phone), (5) stress management, (6) sex, (7) sexual identity (e.g., who an adolescent is attracted to sexually), (8) gender identity, (9) alcohol and drug use, (10) mental health, (11) bullying, and (12) body image. Level of importance for each topic was measured using a 3-point rating scale with response options of: (*1) very important and (2) somewhat important and (3) not at all important*.

### Methods of receiving information

A 7-item measure was developed by research staff to assess who parents would prefer to receive guidance from during a healthcare visit. Parents could choose multiple responses from the following options: (1) pediatrician, (2) nurse, (3) social worker, (4) psychologist, or (5) other. Additionally, a 5-item measure to assess how parents would like to receive guidance during a health care visit was adapted from another study [32]. Parents could choose multiple responses from the following options: (1) written literature, (2) technology, (3) conversation or discussion, (4) group session, or (5) other.

#### Data analysis

The current study used both quantitative and qualitative methods for analyzing results. Descriptive statistics and univariate analyses were used to describe variables of interest. A conventional content analysis approach was used to examine qualitative data and condense open-ended questions into objective categories. Conventional content analysis is often used by collecting data and creating coding schemes, which are later organized into categories or subcategories [33]. Once data collection ended, research staff (LJ, MA, TG) reviewed responses and developed coding schemes to categorize the data. Coding schemes were later condensed and resulted in 4 categories for rewards of parenting and 7 categories for difficulties of parenting (Table 3). All responses were doubled coded. Discrepancies were discussed and reconciled among team members.

## Results

### Demographics

A total of 390 parents were eligible to participate in the study. Research staff were able to contact 229 parents by telephone, and of those, 112 (49%) consented to being in the study. Incomplete survey responses were later withdrawn (*n* = 8; 7%), resulting in a final sample of 104 participants. Parents surveyed had an average age of 41 (SD = 9.6), and the majority were female (93%), African American (70%), and took the survey in English (91%; Table 1). Adolescents who attended the primary care visit with their parent, were, on average, 14 years old (SD = 1.8), African American (74%), and male (52%; Table 1).

**Table 1:** Demographic characteristics (N = 104)

	n	%
Parent Age (M = 41.7 SD = ± 9.6)		
20–29 years old	5	4.8
30–39 years old	45	43.4
40–49 years old	32	30.8
50–59 years old	17	16.2
60–69 years old	3	2.9
70–79 years old	2	1.9
Parent Sex		
Female	97	93.3
Male	7	6.7
Parent Race		
Black	73	70.2
Unreported	13	12.5
White	11	10.6
Multiracial	7	6.7
Parent Ethnicity		
Non-Hispanic or Latinx	84	80.8
Hispanic or Latinx	17	16.3
Unknown/Unreported	3	2.9
Parent Language		
English	95	91.3
Spanish	9	8.7
Adolescent Age (M = 14.5; SD ±1.8)		
12 years old	30	28.8
13 years old	17	16.2
14 years old	15	14.4
15 years old	14	13.8
16 years old	13	12.5
17 years old	11	10.5
18 years old	4	3.8
Adolescent Sex		
Male	54	51.9
Female	50	48.0
Adolescent Race		
Black	77	74.0
White	9	8.7
Unknown/Unreported	18	17.3
Adolescent Ethnicity		
Non-Hispanic or Latinx	86	82.6
Hispanic or Latinx	18	17.3

### Rewards and difficulties of parenting an adolescent

When asked what was the most rewarding part of parenting an adolescent, over half of the parents described adolescent accomplishments and personality characteristics (68%; Table 2). Several parents used words like *respectful, ambitious, loving*, and *smart* when reporting rewarding characteristics of their adolescent*.* Few parents found communication and friendship with their adolescent as rewarding (18%), and some parents stated there was no reward to parenting (2%; Table 2).

**Table 2:** Qualitative analysis of rewards and difficulties of parenting an adolescent (N = 104)

**Rewards of Parenting**			
Key Concept	**n**	**%**	**Representative Quote**
Adolescent Accomplishments and Personality Characteristics	71	68.2	“He′s all around an awesome kid (smart, calm, incredible sense of humor, amazingly loving and caring). Loves looking over his little brother. I love him to death.”
			“She’s a wonderful daughter. She always does her homework. Hangs outside. Is always studying and reading.”
Parenting Benefits	29	27.8	“His rewards that he gets (great student, respectful, smart). Helps me know I’m doing a good job”.
			“Being able to see my child happy and growing up.”
Friendship and Communication	19	18.2	“Well, I like the fact that we have a pretty good relationship. He talks to me easy enough, so the rewarding part is just watching him light up.”
			“The most rewarding part of parenting I would say, maybe, just hanging out with him.”
Nothing/ I Don’t Know	2	1.9	“There is no reward at the moment. The reward will be when she becomes a successful adult.”
			“I don’t know.”
**Difficulties of Parenting**			
Behavioral Issues	38	36.5	“Trying to get him to do the things he is supposed to do. Example, clean up his room.”
			“Sometimes they get to that age where they think they know everything.”
Managing Teen Through Transition Years	24	23.1	“Teenage emotions, hormonal changes, being able to learn how to handle that.”
			“Just the teenage years. Changing of her friends and becoming her own individual person.”
Communication Issues And Difficult Conversations	18	17.3	“Having to explain the real-world things (suicide, sex, drugs). I’d rather be up front and talk to her in person, rather than her use another source like the internet.”
			“Her frustrations. She won’t communicate with me about them.”
Managing parental responsibilities	15	14.4	“Hm. Probably going through the pre-teen years. Finances, being able to afford the things a teen needs. Financial responsibilities of being a parent.”
			“Well, the most difficult part has been making sure he goes to school each day.”
Mental Health	10	9.6	“Problems with anxiety and panic attacks. It is painful and hard to help him manage and figure out what to do. Don’t like seeing him in pain.”
			“Lately he’s been depressed because he misses his dad.”
No difficulties	10	9.6	“I don’t know. He’s a good kid. Nothing difficult.”
			“Nothing. She’s easy to get along with.”

When asked what was the most difficult part of parenting adolescents, some parents described behavioral changes (36%; Table 2). Behavioral changes were often reported by parents as adolescent *attitude, defiance, immaturity*, and *disrespect.* Additionally, some parents reported managing adolescents through transition years as the most difficult part of parenting (23%). Words like *mood swings, changes in hormones,* and *puberty* were frequently used to describe these difficulties. The topic of mental health was described as the least difficult part of parenting (10%), alongside parents who reported no difficulties (10%; Table 2).

### Parenting program and parenting topics

A majority of parents reported communication as a *very important* topic to receive guidance on (*n* = 67; 65%), followed by conflict management (50%), parental monitoring (46%) and discipline (44%; Table 3). Overall, discipline and conflict management were the least important topics to parents. Most parents in the study were interested in participating in a parenting support program available in a primary care-based setting (87%; Table 3).

**Table 3:** Parents level of interest in a parenting program and receiving guidance on parenting and health topics (N = 104)

	n	%
Parents Interest in Participating in a Parenting Program in Primary Care		
Yes	90	86.5
No	13	12.5
Missing	1	1
Parenting Topics Parents Want to Discuss with a Healthcare Provider		
*Communication*		
Very Important	67	65.0
Somewhat Important	19	18.4
Not at all Important	17	16.5
*Conflict Management*		
Very Important	53	50.9
Somewhat Important	32	30.7
Not at all Important	19	18.2
*Parental Monitoring*		
Very Important	48	46.1
Somewhat Important	25	24.0
Not at all Important	31	29.8
*Discipline*		
Very Important	46	44.2
Somewhat Important	27	25.9
Not at all Important	31	29.8
Health Topics Parents Want to Discuss with a Healthcare Provider		
*Sex*		
Very Important	80	77.6
Somewhat Important	12	11.6
Not at all Important	11	10.6
*Mental Health*		
Very Important	78	75.0
Somewhat Important	20	19.2
Not at all Important	6	5.7
*Alcohol and Drugs*		
Very Important	77	74.0
Somewhat Important	11	10.5
Not at all Important	16	15.3
*Stress Management*		
Very Important	75	72.1
Somewhat Important	20	19.2
Not at all Important	9	8.6
*Bullying*		
Very Important	74	71.5
Somewhat Important	18	17.3
Not at all Important	12	11.5
*Sexual Identity*		
Very Important	66	63.4
Somewhat Important	15	14.4
Not at all Important	23	22.1
*Body Image*		
Very Important	66	63.4
Somewhat Important	23	22.1
Not at all Important	15	14.4
*Monitoring Internet Use*		
Very Important	60	57.6
Somewhat Important	19	18.2
Not at all Important	25	24.0
*Romantic Relationships*		
Very Important	59	56.7
Somewhat Important	25	24.0
Not at all Important	20	19.2
*Gender Identity*		
Very Important	58	55.7
Somewhat Important	19	18.2
Not at all Important	27	25.9
*Friendships*		
Very Important	52	50.0
Somewhat Important	30	28.8
Not at all Important	22	21.1
*Mobile Technology*		
Very Important	49	47.1
Somewhat Important	26	25.0
Not at all Important	29	27.8

### Health topics

Of the 12 health topics listed, a large number of parents rated sex as a *very important* topic to receive guidance on from a HCP (*n* = 80; 77%); however, mental health (75%) and alcohol and drugs were in close proximity (74%; Table 3). By contrast, the topic of mobile technology was rated highest in the category of *not at all important* (27%; Table 3).

### Methods for receiving guidance

A majority of parents reported a pediatrician as the most suitable person to provide parenting and health topic guidance (*parenting topics* = 62%; *health topics* = 66%; Table 4). When asked how to deliver information, parents frequently reported a preference for written literature (e.g., book or pamphlet; *parenting topic* = 53%; *health topic* = 50%), followed by the use of technology (e.g., phone app or referral to website; *parenting topic* = 39%; *health topic* = 44%; Table 4).

**Table 4:** Methods for which parents want to receive guidance (N = 104)

	n	%
Who do Parents Want to Talk with About Parenting Topics?		
Pediatrician	64	61.5
Psychologist	24	23.0
Social Worker	22	21.1
Nurse	15	14.4
Community Partner	7	6.7
Family	3	2.8
No Preference	2	1.9
How do Parents Want to Receive Guidance on Parenting Topics?		
Written Literature	55	53
Technology	41	39
Conversation/Discussion	16	15
Group Session	9	9
Other	1	1
Who do Parents Want to Talk with About Adolescent Health Topics?		
Pediatrician	69	66.3
Psychologist	22	21.1
Nurse	17	16.3
Social Worker	9	8.6
Community Partner	7	6.7
Family	5	4.8
Other	3	2.8
How do Parents Want to Receive Guidance on Adolescent Health Topics?		
Written Literature	52	50
Technology	46	44
Conversation/Discussion	20	19
Group Session	3	3
Other	2	2

## Discussion

Parental guidance and parenting programs are supported and recommended by healthcare organizations [11, 34]; however, few studies have investigated parents’ perspectives on receiving parenting guidance in healthcare settings. Our results demonstrated that parents are interested in speaking to HCPs on parenting topics including how to communicate with their adolescent and how to manage parent-adolescent conflicts. Parents also reported being interested in receiving advice on discipline and parental monitoring. Additionally, more parents were inclined to receiving parental guidance and support from pediatricians as compared to other HCPs, community leaders, and family members. The fact that pediatricians were considered trusted providers of information is important to note, as our sample was primarily African American parents and medical mistrust tends to be higher among this community [35, 36]. Recent studies have suggested that a key contributor to strengthening trust between African Americans and HCPs is by increasing communication, time, and energy spent together during healthcare visits [37, 38]. Thus, one way to strengthen the relationship between the African American and medical community could be by pediatricians providing parenting support and anticipatory guidance during primary care visits. Although more research is needed to support such a claim, our results do reinforce that parents (in general) are seeking advice beyond *health* topics and are open and willing to receive *parenting* support from HCPs.

Results from our study also found that over 70% of parents were interested in receiving guidance on topics of sex, mental health, stress management, bullying, body image, and alcohol and drugs. Other studies have reported similar findings, [14, 16] and our qualitative data further support these results. For instance, parents described difficult conversations (e.g., discussing sex, alcohol, and drugs) as a challenging aspect of parenting. Another example can be found in parents’ reported descriptions of assisting their adolescent through the transitional years (e.g., puberty, hormones, and identity change) and wanting guidance from HCPs on the topic.

Interestingly, few parents described communication as a difficult part of parenting (17%), and yet 83% listed communication as a very important *parenting* topic to discuss with a HCP. Additionally, few parents described mental health as a difficult part of parenting (10%), and yet 75% of parents rated it as a very important adolescent *health* topic. Although it is unclear why few parents reported communication and mental health as a difficult part of parenting, and then later reported both as very important topics to receive guidance on, one explanation could be that parents were hesitant to address the challenges they faced while raising their adolescent. For example, given the increasing rise of adolescent depression and suicide, [39] parents may have been disinclined to admit that their adolescents’ mental health was a concern or issue due to fear of stigma or perceived parent ineffectiveness. Another possible explanation is that parents were not familiar with the signs or symptoms of certain mental health disorders, which could explain why a large proportion of the parents wanted advice from HCPs on mental health. Despite these conflicting results, several parents described watching their child grow and achieve accomplishments as a rewarding part of parenting.

In addition to our findings listed above, our study also found that parents from our sample were interested in a parenting program in a primary care-based setting (87%). Despite the presence of established parenting programs in non-medical settings, [18, 19] implementing similar programs in healthcare is desirable from the perspectives of parents. Previous implementation studies have assessed the integration of more general parent-family education programs into healthcare settings, [13, 34] and recommendations for implementing these programs include increasing parent-HCP direct communication, providing opportunities for weekly or monthly parenting groups, and as our study reinforced, through written forms of communication and technology resources [9, 40].

Technology resources, online treatment modules, and social networking tools could be offered by HCPs, [41] and monthly newsletters, pamphlets, or books could be distributed throughout clinics. Additionally, technology resources - such as electronic screeners - could be developed to evaluate parenting needs prior to their adolescent’s healthcare visit, and HCPs could then target those needs during the healthcare appointment. Considering the added costs and restricted time HCPs have with families, using technology-focused tools could also be used to provide formal training to reduce the time, money and energy required for in-person training workshops. More importantly, technology focused tools would also allow other HCPs (e.g., psychologist, social worker, nurse) to receive formal training and furnish primary care clinics with diverse, multidisciplinary methods of assisting parents.

Although the present study highlighted various methods for improving parents’ knowledge in the context of primary care-based settings, our results should be interpreted with caution, as there were several limitations. One limitation of our study is that parents were recruited from clinics in one urban, county healthcare system and were primarily female (93%) and African American (70%), resulting in unbalanced sex and race ratios. Non-female parents or those of other races/ethnicities may have had different parenting needs. Therefore, the results of this study have limited external validity, but may be appropriate to similar settings. Second, our study did not gather data on the household size or whether parents had any previous experience with raising an adolescent. The support needs of parents raising their first adolescent child may not mirror the needs of parents with multiple children past the age of adolescence. Thus, some data that were captured may not accurately represent all parents’ needs. Third, our study resulted in a small sample size (104 parents). However, given the time restraints to contact parents (48-h recruitment window and business hour availability), it is understandable why many of our eligible parents were not available to take the survey. Fourth, although parents were informed that our study was not associated with the hospital system or primary care clinic in which their child received services, parents may still have responded to the questions with a social-desirability bias. Lastly, in spite of parents’ interest in parenting programs, it is unclear whether HCPs have the time and needed expertise to provide parental support. Providing such programs would be costly, require negotiations with health insurers, and entail HCPs to receive additional training.

## Conclusion

Our study found that despite the rewarding aspects of raising adolescents, parents identify a handful of difficulties associated with parenting, and thus report interest in receiving parental guidance and support from HCPs in primary care settings. HCPs have the potential to fulfill these needs. Research has shown that the implementation of parenting programs in non-medical settings are feasible and effective, and that parents are interested in expanding this to healthcare settings [18, 19]. Given that strategies for implementing other parenting programs within primary care-based settings presently exist, [13, 34] it would be helpful for primary care clinics to likewise dedicate resources to supporting the parents of adolescents.

## Supplementary Information

**Additional file 1.**

## Data Availability

The datasets used and/or analyzed during the current study are available from the corresponding author on reasonable request.

## Abbreviations

HCP: Healthcare Provider
HCPs: Healthcare Providers
AAP: American Academy of Pediatrics
CDSS: Clinical Decision Support System
CHICA: Child Health Improvement through Computer Automation System
PResNet: Pediatric Research Network
