# Supplementary material for: How can healthcare professionals provide guidance and support to parents of adolescents? Results from a primary care-based study
Source: BMC Health Serv Res. 2021 Mar 20;21:253. doi: 10.1186/s12913-021-06200-1 (PMC7981794; doi:10.1186/s12913-021-06200-1)
Supplement: Supplementary file 1 — Additional file 1. [file 12913_2021_6200_MOESM1_ESM.pdf]

96	scrpt8	Section<br>Header: *DEMOGRAPHICS*<br><br>RA SCRIPT: First I'll ask a couple of questions about you.<br><br>Primero le formularé algunas preguntas sobre usted.	descriptive
97	crgvr_age	What is your age?<br><br>¿Qué edad tiene?	text
98	crgvr_ethncty	Do you consider yourself Hispanic or Latino/a?<br><br>¿Se considera hispano(a) o latino(a)?	radio<br><br>1 Hispanic or Latino / Es hispano(a) o latino(a)<br>2 Non Hispanic or Latino / Ni hispano(a) ni latino(a)<br>3 Unknown or Unreported / Lo desconoce o No informó
99	crgvr_race	What is your race?<br><br>¿Cuál es su raza?	radio<br><br>1 Asian / Asiática

<table>
<tr><td></td><td></td><td></td><td><table>
<tr><td>2</td><td>American Indian or Alaskan Native / India americana o Nativa de Alaska</td></tr>
<tr><td>3</td><td>Native Hawaiian or Pacific Islander / Nativa de Hawái o de las islas del Pacífico</td></tr>
<tr><td>4</td><td>Black / Negra</td></tr>
<tr><td>5</td><td>Caucasian or White / Caucásica o Blanca</td></tr>
<tr><td>6</td><td>More than one race / Más de una raza</td></tr>
<tr><td>7</td><td>Unknown or Unreported / La desconoce o No informó</td></tr>
</table></td></tr>
<tr><td>100</td><td>scrpt9</td><td>RA SCRIPT: Thank you for taking the time to answer the questions on the tablet computer before seeing the healthcare provider.

Gracias por tomarse el tiempo de responder las preguntas en la computadora tipo tableta antes de ver al proveedor de atención de salud.
RA NOTE: For any open-ended questions, try to record the caregiver's response verbatim.</td><td>descriptive</td></tr>
</table>

101	scrpt10<br><br>Show the field ONLY if:<br><br>[slf_rprt_used_tblt] = '1'	RA SCRIPT: Now I would like to ask you some general questions about being a parent or caregiver to [ptnt_fn].<br><br>Ahora me gustaría formularle algunas preguntas generales como padre/madre o cuidador de [ptnt_fn].	descriptive
102	crgvr_mst_rwrdng_prt_text<br><br>Show the field ONLY if:<br><br>[slf_rprt_used_tblt] = '1'	RA SCRIPT: What is the most rewarding part of parenting [ptnt_fn]?<br><br>¿Qué es lo más gratificante de ser padre/madre de [ptnt_fn]?	notes
103	crgvr_mst_dfclt_prt_text<br><br>Show the field ONLY if:<br><br>[slf_rprt_used_tblt] = '1'	RA SCRIPT: What has been the most difficult part of parenting [ptnt_fn]?<br><br>¿Cuál ha sido la parte más difícil de ser padre/madre de [ptnt_fn]?	notes<br>Custom alignment: LH
104	crgvr_teen_hlth_prvd_rltnshp_rmntc<br><br>Show the field ONLY if:<br><br>[slf_rprt_used_tblt] = '1'	Section Header: *RA SCRIPT: Now I'm going to list some common teen health topics that you may want to discuss with a pediatrician or other healthcare provider about*	radio (Matrix)<br><br>1 | Not at all important No es importante<br>2 | Somewhat important Es algo importante

		[ptnt_fn]. Let me know if you would say the topic is not at all important, somewhat important, or very important to you. In your opinion, how important would it be for [ptnt_fn]'s pediatrician to talk to you about: Ahora voy a enumerar algunos temas de salud comunes de los adolescentes que usted probablemente desee conversar con un pediatra u otro proveedor de atención de salud sobre de [ptnt_fn]. Dígame si considera que el tema no es importante, es algo importante o es muy importante para usted. En su opinión, ¿qué tan importante sería que un pediatra de [ptnt_fn] hablara con usted sobre:<br><br>How to provide [ptnt_fn] relationship advice regarding romantic partners Cómo proporcionar [ptnt_fn] orientación en la relación con parejas románticas	3 Very important Es muy importante

105	crgvr_teen_hlth_prvd_rltnshp_frnds<br><br>Show the field ONLY if:<br><br>[slf_rprt_used_tblt] = '1'	How to provide [ptnt_fn] relationship advice regarding friends Cómo proporcionar [ptnt_fn] orientación en la relación con los amigos?	radio (Matrix)<br><br>1 Not at all important No es importante<br>2 Somewhat important Es algo importante<br>3 Very important Es muy importante
106	crgvr_teen_hlth_mntr_intrnt_use<br><br>Show the field ONLY if:<br><br>[slf_rprt_used_tblt] = '1'	How to monitor [ptnt_fn]'s internet use Cómo monitorizar el uso del [ptnt_fn] del internet?	radio (Matrix)<br><br>1 Not at all important No es importante<br>2 Somewhat important Es algo importante<br>3 Very important Es muy importante
107	crgvr_teen_hlth_strtgs_mbl_tchnlgy_use<br><br>Show the field ONLY if:<br><br>[slf_rprt_used_tblt] = '1'	Strategies around [ptnt_fn]'s use of mobile technology (i.e., cell phone use) Estrategias sobre el uso de [ptnt_fn] de la tecnología móvil (es decir, uso del teléfono celular)?	radio (Matrix)<br><br>1 Not at all important No es importante<br>2 Somewhat important Es algo importante<br>3 Very important Es muy importante
108	crgvr_teen_hlth_how_aid_strs_mngmnt<br><br>Show the field ONLY if:<br><br>[slf_rprt_used_tblt] = '1'	How to aide [ptnt_fn] in stress management Cómo ayudar [ptnt_fn] en el manejo del estrés?	radio (Matrix)<br><br>1 Not at all important No es importante<br>2 Somewhat important Es algo importante

			3 Very important Es muy importante
109	crgvr_teen_hlth_advc_tlkng_sex<br>Show the field ONLY if:<br>[slf_rprt_used_tblt] = '1'	Advice about talking to [ptnt_fn] about sex Orientación sobre cómo hablar con [ptnt_fn] de sexo?	radio (Matrix)<br>1 Not at all important No es importante<br>2 Somewhat important Es algo importante<br>3 Very important Es muy importante
110	crgvr_teen_hlth_advc_tlkng_sxl_idnty<br>Show the field ONLY if:<br>[slf_rprt_used_tblt] = '1'	Advice about talking to [ptnt_fn] about sexual identity (i.e. who they are attracted to sexually) Orientación sobre cómo hablar con [ptnt_fn] de la identidad sexual (es decir, hacia quiénes se siente atraído sexualmente)?	radio (Matrix)<br>1 Not at all important No es importante<br>2 Somewhat important Es algo importante<br>3 Very important Es muy importante
111	crgvr_teen_hlth_advc_tlkng_gndr_idnty<br>Show the field ONLY if:<br>[slf_rprt_used_tblt] = '1'	Advice about talking to [ptnt_fn] about gender identity (i.e. their internal sense of being male, female or combination of both)Orientación sobre cómo hablar con [ptnt_fn] de la identidad del género (es decir, su sentido interno de ser varón, mujer o una combinación de ambos)?	radio (Matrix)<br>1 Not at all important No es importante<br>2 Somewhat important Es algo importante<br>3 Very important Es muy importante

112	crgvr_teen_hlth_advc_tlkng_alchl_drgs<br>Show the field ONLY if:<br>[slf_rprt_used_tblt] = '1'	Advice about talking to [ptnt_fn] about alcohol and drugsOrientación sobre cómo hablar con [ptnt_fn] del alcohol y las drogas?	radio (Matrix)<br>1 Not at all important No es importante<br>2 Somewhat important Es algo importante<br>3 Very important Es muy importante
113	crgvr_teen_hlth_advc_tlkng_mntl_hlth<br>Show the field ONLY if:<br>[slf_rprt_used_tblt] = '1'	Advice about talking to [ptnt_fn] about mental health issues Orientación sobre cómo hablar con [ptnt_fn] de los problemas de salud mental?	radio (Matrix)<br>1 Not at all important No es importante<br>2 Somewhat important Es algo importante<br>3 Very important Es muy importante
114	crgvr_teen_hlth_advc_tlkng_bdy_img<br>Show the field ONLY if:<br>[slf_rprt_used_tblt] = '1'	Advice about talking to [ptnt_fn] about body imageOrientación sobre cómo hablar con [ptnt_fn] de la imagen corporal?	radio (Matrix)<br>1 Not at all important No es importante<br>2 Somewhat important Es algo importante<br>3 Very important Es muy importante
115	crgvr_teen_hlth_how_intrvn_blyng<br>Show the field ONLY if:<br>[slf_rprt_used_tblt] = '1'	How to intervene regarding bullying Cómo intervenir en relación con la intimidación/acoso?	radio (Matrix)<br>1 Not at all important No es importante<br>2 Somewhat important Es algo importante

			3 | Very important Es muy importante
116	crgvr_teen_hlth_othr_info_wntd<br><br>Show the field ONLY if:<br><br>[slf_rprt_used_tblt] = '1'	RA SCRIPT: What other health information would be helpful to get from a pediatrician or other healthcare provider regarding [ptnt_fn]?<br><br>¿Qué otra información de salud sería útil obtener de un pediatra u otro proveedor de atención de salud en relación con [ptnt_fn]?	notes<br>Custom alignment: LH
117	crgvr_teen_hlth_who_spk_abt_tpcs<br><br>Show the field ONLY if:<br><br>[slf_rprt_used_tblt] = '1'	RA SCRIPT: As a parent, who would you want to speak to you about the above health topics regarding [ptnt_fn] (i.e. nurse, social worker, psychologist)?<br><br>Como padre/madre, ¿con quién desearía hablar sobre los temas de salud en relación con [ptnt_fn] (es decir, enfermera, trabajador social, psicólogo)?	checkbox<br><br>1 | crgvr_teen_hlth_who_spk_abt_tpcs___1 | Pediatrician / Pediatra<br>2 | crgvr_teen_hlth_who_spk_abt_tpcs___2 | Nurse / Enfermera<br>3 | crgvr_teen_hlth_who_spk_abt_tpcs___3 | Social Worker / Trabajadora social<br>4 | crgvr_teen_hlth_who_spk_abt_tpcs___4 | Psychologist / Psicólogo

<table>
<tr><td></td><td></td><td></td><td><table><tr><td>5</td><td>crgvr_teen_hlth_who_spk_abt_tpcs___5</td><td>Other</td></tr></table>
Custom alignment: LV</td></tr>
<tr><td>118</td><td>crgvr_teen_hlth_who_spk_abt_tpcs_othr
Show the field ONLY if:
[crgvr_teen_hlth_who_spk_abt_tpcs(5)] = '1'</td><td>Other person they want to provide information:</td><td>text
Custom alignment: RV</td></tr>
<tr><td>119</td><td>crgvr_teen_hlth_how_get_info_tpcs
Show the field ONLY if:
[slf_rprt_used_tblt] = '1'</td><td>RA SCRIPT: In addition to talking with [ptnt_fn]'s pediatrician or another healthcare provider, how would you like to get information like this (i.e. receiving an email from a provider) or (i.e. referral to a book)?

Además de hablar con el pediatra de [ptnt_fn] u otro proveedor de atención salud, ¿cómo le gustaría obtener información como esta (i.e. recibiendo un email de un proveedor o referencia a un libro)?</td><td>checkbox
<table><tr><td>1</td><td>crgvr_teen_hlth_how_get_info_tpcs___1</td><td>Handout given at visit / Folleto entregado durante la visita</td></tr><tr><td>2</td><td>crgvr_teen_hlth_how_get_info_tpcs___2</td><td>Website / Sitio web</td></tr><tr><td>3</td><td>crgvr_teen_hlth_how_get_info_tpcs___3</td><td>Apps / Aplicaciones</td></tr><tr><td>4</td><td>crgvr_teen_hlth_how_get_info_tpcs___4</td><td>Text Messaging /</td></tr></table></td></tr>
</table>

			| | | Mensajería de texto | | 5 | crgvr_teen_hlth_how_get_info_tpcs___5 | Other / Otro | <br>Custom alignment: LV
120	crgvr_teen_hlth_how_get_info_tpcs_othr<br><br>Show the field ONLY if:<br><br>[crgvr_teen_hlth_how_get_info_tpcs(5)] = '1'	Other way of receiving information:	text<br>Custom alignment: RV
121	crgvr_prntng_cnflct_mngmnt<br><br>Show the field ONLY if:<br><br>[slf_rprt_used_tblt] = '1'	Section Header: *RA SCRIPT: Now I'm going to list some common parenting topics that you may want to discuss with a pediatrician or other healthcare provider. Let me know if you would say the topic is not at all important, somewhat important, or very important to you? In your opinion, how important would it be for [ptnt_fn]'s pediatrician to talk to you about: Ahora voy a enumerar algunos temas comunes sobre la crianza que es posible que*	radio (Matrix)<br><br>| 1 | Not at all important No es importante | | 2 | Somewhat important Es algo importante | | 3 | Very important Es muy importante |

		desee tratar con un pediatra u otro proveedor de atención de salud. Dígame si considera que el tema no es importante, es algo importante o es muy importante para usted. En su opinión, ¿qué tan importante sería que la pediatra de [ptnt_fn] hablara con usted sobre:<br><br>Conflict management (e.g., resolving fights between parent and teen)Manejo de conflictos (p. ej.: resolver las peleas entre los padres y los adolescentes)	
122	crgvr_prntng_dscpln<br><br>Show the field ONLY if:<br><br>[slf_rprt_used_tblt] = '1'	Discipline (e.g., punishing teen for inappropriate behavior) Disciplina (p. ej.: castigar al adolescente por conducta inapropiada)	radio (Matrix)<br><br>1 Not at all important No es importante<br>2 Somewhat important Es algo importante<br>3 Very important Es muy importante
123	crgvr_prntng_prntl_mntrng<br><br>Show the field ONLY if:<br><br>[slf_rprt_used_tblt] = '1'	Parental monitoring (e.g., knowing where [ptnt_fn] is after school; knowing [ptnt_fn]'s friends)Monitorización parental (p. ej.: saber dónde	radio (Matrix)<br><br>1 Not at all important No es importante<br>2 Somewhat important Es algo importante

		está [ptnt_fn] después de salir de la escuela; conocer a sus amigos)	3 Very important Es muy importante
124	crgvr_prntng_cmnctng<br>Show the field ONLY if:<br>[slf_rprt_used_tblt] = '1'	Communicating with [ptnt_fn] Comunicación con [ptnt_fn]	radio (Matrix)<br>1 Not at all important No es importante<br>2 Somewhat important Es algo importante<br>3 Very important Es muy importante
125	crgvr_prntng_othr_info_wntd<br>Show the field ONLY if:<br>[slf_rprt_used_tblt] = '1'	RA SCRIPT: What other parenting information would be helpful to get from a pediatrician or other healthcare provider regarding [ptnt_fn]?<br><br>¿Qué otra información de la crianza sería útil obtener de un pediatra u otro proveedor de atención de salud en relación con [ptnt_fn]?	notes<br>Custom alignment: LH
126	crgvr_prntng_who_spk_abt_tpcs<br>Show the field ONLY if:<br>[slf_rprt_used_tblt] = '1'	RA SCRIPT: As a parent, who would you want to speak to you about the above parenting topics regarding [ptnt_fn] (i.e. nurse, social worker, psychologist)?	checkbox<br>1 crgvr_prntng_who_spk_abt_tpcs___1 Pediatrician / Pediatra

		Como padre/madre, ¿con quién desearía hablar sobre los temas de la crianza en relación con [ptnt_fn] (es decir, enfermera, trabajador social, psicólogo)?	2 | crgvr_prntng_who_spk_abt_tpcs___2 | Nurse / Enfermera<br>3 | crgvr_prntng_who_spk_abt_tpcs___3 | Social Worker / Trabajadora social<br>4 | crgvr_prntng_who_spk_abt_tpcs___4 | Psychologist / Psicólogo<br>5 | crgvr_prntng_who_spk_abt_tpcs___5 | Other<br><br>Custom alignment: LV
127	crgvr_prntng_who_spk_abt_tpcs_othr<br><br>Show the field ONLY if:<br><br>[crgvr_prntng_who_spk_abt_tpcs(5)] = '1'	Other person they want to provide information:	text<br>Custom alignment: RV
128	crgvr_prntng_how_get_info_tpcs<br><br>Show the field ONLY if:<br><br>[slf_rprt_used_tblt] = '1'	RA SCRIPT: In addition to talking with [ptnt_fn]'s pediatrician or another healthcare provider, how would you like to get information like this (i.e. receiving an email from a provider) or (i.e. referral to a book)?	checkbox<br><br>1 | crgvr_prntng_how_get_info_tpcs___1 | Handout given at visit / Folleto entregado durante la visita

<table>
<tr><td></td><td></td><td>Además de hablar con el pediatra de [ptnt_fn] u otro proveedor de atención salud, ¿cómo le gustaría obtener información como esta (i.e. recibiendo un email de un proveedor o referencia a un libro)?</td><td><table><tr><td>2</td><td>crgvr_prntng_how_get_info_tpcs___2</td><td>Website / Sitio web</td></tr><tr><td>3</td><td>crgvr_prntng_how_get_info_tpcs___3</td><td>Apps / Aplicaciones</td></tr><tr><td>4</td><td>crgvr_prntng_how_get_info_tpcs___4</td><td>Text Messaging / Mensajería de texto</td></tr><tr><td>5</td><td>crgvr_prntng_how_get_info_tpcs___5</td><td>Other / Otro</td></tr></table>
Custom alignment: LV</td></tr>
<tr><td>129</td><td>crgvr_prntng_how_get_info_tpcs_othr
Show the field ONLY if:
[crgvr_prntng_how_get_info_tpcs(5)] = '1'</td><td>Other way of receiving information:</td><td>text
Custom alignment: RV</td></tr>
<tr><td>130</td><td>lastly</td><td>RA SCRIPT: Lastly, we are planning a program for parents of teens, which will be focused on addressing some of these issues in pediatric visits. Would you be interested in participating in the program?</td><td>Yes no<table><tr><td>1</td><td>Yes</td></tr><tr><td>0</td><td>No</td></tr></table>
Custom alignment: LH</td></tr>
</table>

		Por último, estamos planificando un programa para padres/madres de adolescentes, que estará orientado a atender algunos de estos problemas en las visitas pediátricas. ¿Le interesaría participar en el programa?	
